# Supplementary figures and images for: Identification of PIMREG as a novel prognostic signature in breast cancer via integrated bioinformatics analysis and experimental validation
Source: PeerJ. 2023 Jul 17;11:e15703. doi: 10.7717/peerj.15703 (PMC10358341; doi:10.7717/peerj.15703)

Repeat1

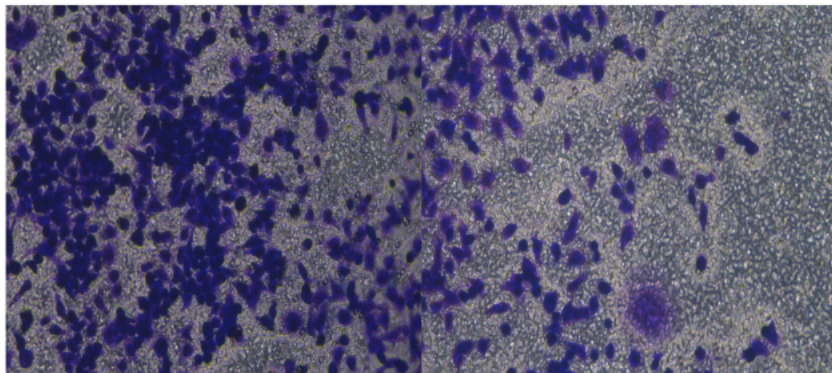

Con

siPIMREG

Repeat2

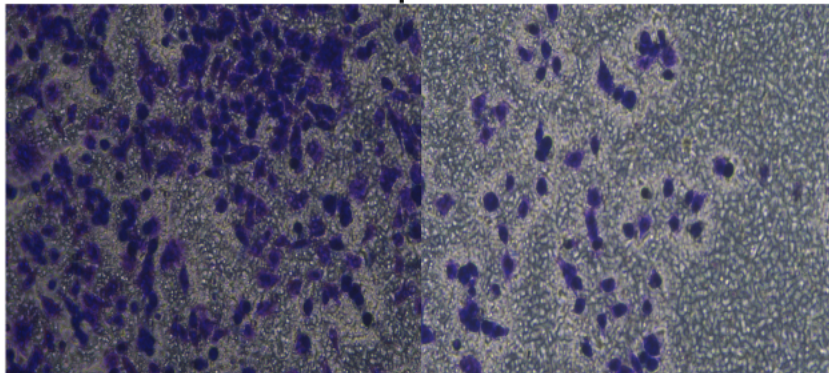

Repeat3

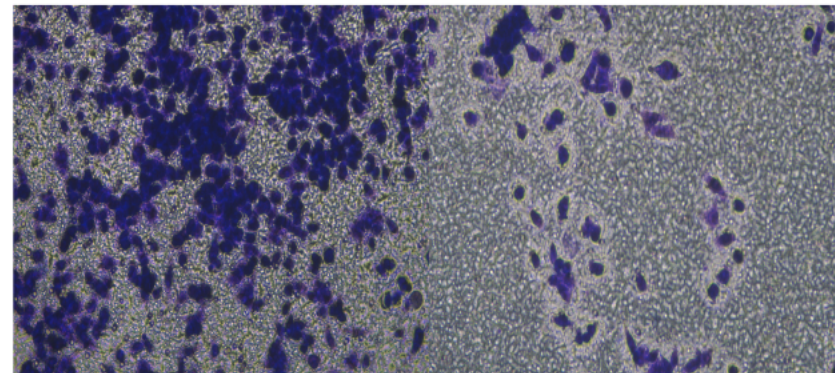

Supplement: Supplemental Information 1 [file peerj-11-15703-s001.pdf]

Repeat1

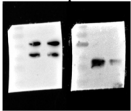

Repeat2

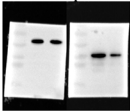

Repeat3

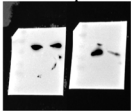

Actin PIMREG

Supplement: Supplemental Information 2 [file peerj-11-15703-s002.pdf]

Repeat1

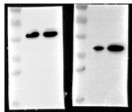

actin PIMREG

Repeat2

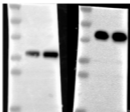

PIMREG actin

Repeat3

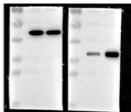

actin PIMREG

Supplement: Supplemental Information 3 [file peerj-11-15703-s003.pdf]

P1

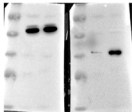

P2

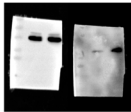

P3

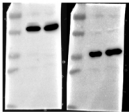

P4

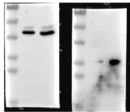

P5

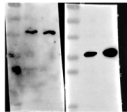

actin PIMREG actin PIMREG actin PIMREG actin PIMREG actin PIMREG

Supplement: Supplemental Information 4 [file peerj-11-15703-s004.pdf]
